# Histological Features of the Gastrointestinal Tract of Wild Indonesian Shortfin Eel, *Anguilla bicolor bicolor* (McClelland, 1844), Captured in Peninsular Malaysia

**DOI:** 10.1155/2014/312670

**Published:** 2014-12-23

**Authors:** Nurrul Shaqinah Nasruddin, Mohammad Noor Amal Azmai, Ahmad Ismail, Mohd Zamri Saad, Hassan Mohd Daud, Syaizwan Zahmir Zulkifli

**Affiliations:** ^1^Department of Veterinary Laboratory Diagnostics, Faculty of Veterinary Medicine, Universiti Putra Malaysia (UPM), 43400 Serdang, Selangor, Malaysia; ^2^Department of Biology, Faculty of Science, Universiti Putra Malaysia (UPM), 43400 Serdang, Selangor, Malaysia

## Abstract

This study was conducted to record the histological features of the gastrointestinal tract of wild Indonesian shortfin eel, *Anguilla bicolor bicolor* (McClelland, 1844), captured in Peninsular Malaysia. The gastrointestinal tract was segmented into the oesophagus, stomach, and intestine. Then, the oesophagus was divided into five (first to fifth), the stomach into two (cardiac and pyloric), and the intestine into four segments (anterior, intermediate, posterior, and rectum) for histological examinations. The stomach had significantly taller villi and thicker inner circular muscles compared to the intestine and oesophagus. The lamina propria was thickest in stomach, significantly when compared with oesophagus, but not with the intestine. However, the intestine showed significantly thicker outer longitudinal muscle while gastric glands were observed only in the stomach. The histological features were closely associated with the functions of the different segments of the gastrointestinal tract. In conclusion, the histological features of the gastrointestinal tract of *A. b. bicolor* are consistent with the feeding habit of a carnivorous fish.

## 1. Introduction

The Indonesian shortfin eel,* Anguilla bicolor bicolor* (McClelland, 1844), is native to Malaysia. Nevertheless, the presence of* A. b. bicolor* has been reported in different geographical locations throughout the world, such as Greater Sunda Islands (Java and Sumatra in western Indonesia), north-western Australia, Africa, Myanmar, India, Sri Lanka, Bangladesh, and Malaysia [1–3]. In Malaysia,* A. b. bicolor* are found in estuarine areas and they occasionally migrate around 60 km from the ocean into freshwater rivers [3]. This is due to the migration behavior of the eels as well as a strategy to avoid competition for space and food [4, 5].

The European eels,* A. anguilla*, have a broad diet and their primary food source is aquatic invertebrates, but sometimes they will eat any food they find including dead organisms [6]. The feeding habits of* A. anguilla* consisted of benthic organisms, primarily amphipod crustaceans, polychaetes, insect larvae, molluscs, and small fishes [7]. For* A. bicolor*, a study showed that the fish change their feeding habits markedly as they grow. They feed mainly on invertebrates when small and become more piscivorous as they grow, while no seasonal variation in the feeding habits was observed [8]. Their main feeding habits consisted of macrophytes, algae, crustaceans, mollusks, insects, annelids, insect larvae, and bony fishes [8–10].

Gastrointestinal tract of fish shows marked diversity in morphology and function, which is useful to determine the taxonomy, feeding habits, and habitat [11, 12]. Indeed, each segment of the digestive tract has a mucosal specialization for an optimal efficiency of secretion, absorption, and digestive functions [13]. Therefore, the main function of fish intestine is to complete the digestive process, which started in the stomach, and to absorb the nutrients from food [14]. The gross and histological features of gastrointestinal tract of fishes were also different based on taxonomy, feeding habit, and body shape [15]. The morphological features of gastrointestinal tract of carnivorous fish were proved to diverge from herbivorous fish. The carnivorous fish tend to have shorter intestine than the herbivorous fish due to lower percentage of plant materials in the diet [16, 17]. Histologically, the oesophagus of a carnivorous fish has more mucous cells than herbivorous fish [18]. For stomach of a carnivorous fish,* Schilbe mystus*, a study found that it can be divided into two compartments, namely, cardiofundic and pyloric, although the organ morphologically consisted of three parts, which are cardiac, pyloric, and fundic region [19]. In addition, the gastric mucosa of perch,* Perca fluviatilis*, was described to have three types of endocrine cells that produced hydrochloric acid [20], but it is absent in herbivorous fish such as sea garfish,* Hyporhamphus melanochir* [21]. The mucosa of the intestine of* S. mystus* consisted of simple columnar epithelium that contains Goblet cells, similar to the histological structure in herbivorous fish,* Labeo niloticus* [22].

There are limited studies on the histological features of digestive tract of* Anguilla*, while most of the conducted studies in this country focused more on the inventory survey and migration behavior of the eels [2, 3]. Thus, the present study was conducted in order to describe the histological features of the gastrointestinal tract, particularly the oesophagus, stomach, and intestine of wild* A. b. bicolor*, captured in Peninsular Malaysia. This may provide a comparative basis for future studies of digestion, absorption, and feeding pattern. In addition, the ultrastructural analysis described from this study is important for the diagnosis of diseases related to gastrointestinal tract of* Anguilla*.

## 2. Materials and Methods

*Anguilla bicolor bicolor* samples were captured in Balik Pulau, Penang, Malaysia, from February 2013 to January 2014. A total of 28 wild eel samples were obtained either by angling or by using traditional fish traps. Following capture, the wet body mass and total length were measured and recorded.

Samples were placed in a solution containing tricaine methanesulfonate (MS 222) at 50 mg/L to achieve stage three of anesthesia, before they were sacrificed by cervical dislocation according to method approved by the Animal Utilization Protocol, Universiti Putra Malaysia. The entire digestive tract was immediately extracted by a midventral incision, removed, and then divided into oesophagus, stomach, and intestine.

Anatomical description was carried out before the oesophagus, stomach, and intestine were divided into five (first to fifth), two (cardiac and pyloric), and four (anterior, intermediate, posterior, and rectum) segments, respectively [23]. Organs were then sampled in triplicate, labeled, and fixed in 10% buffered formalin for 12 hours before they were embedded in paraffin, sectioned at 5 *μ*m thick, and stained routinely with Harris haematoxylin and eosin (HE) and periodic acid-Schiff (PAS). The height of villi, thickness of lamina propria, inner circular muscle, outer longitudinal muscle, and gastric gland were measured according to Firdaus-Nawi et al. [24]. The photos were recorded through photomicroscope (Nikon Eclipse 50i, Japan) and analyzed through The Nikon NIS-Element D 3.2 Image Analyser (Nikon Instruments Inc., USA).

The data was checked for normality using Shapiro-Wilk test of normality (*P* > 0.05) and its homogeneity of variances using Levene's test for equality of variances (*P* > 0.05) (IBM SPSS Statistics Version 21). The mean ± standard error of mean (SEM) of the histological measurements for each structure was compared among all of the segments and organs using the analysis of variance with Tukey LSD All-Pairwise Comparison Test (Statistix 9, Analytical Software) [25]. The significance value was at *P* < 0.05. The determined measurements were the height for the mucosal projection and the thickness for lamina propria, inner circular muscle, outer longitudinal muscle, and gastric gland.

## 3. Results

The mean (±standard deviations) of wet body mass and total length of the 28 samples of* A. b. bicolor* were 378.47 (±246.78) g and 560.53 (±140.93) mm, respectively.

Table 1 shows the measurements of villus height, thickness of lamina propria, inner circular muscle, outer longitudinal muscle, and gastric gland of the sampled* A. b. bicolor*. The stomach showed significantly higher villous and thicker inner circular muscle than the intestine and oesophagus. The lamina propria was the thickest in the stomach and significantly thicker than in oesophagus but not than in the intestine. The intestine showed significantly thicker outer longitudinal muscle than oesophagus and stomach. The gastric glands were observed only in the stomach.

### 3.1. Histological Features of the Oesophagus

**Figure 1:**
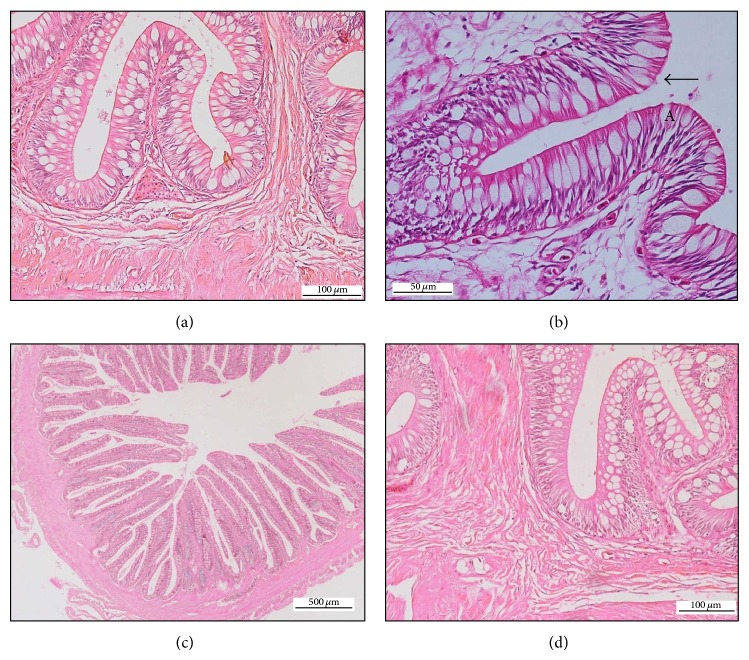
(a) Goblet cells at the mucosal surface of oesophagus, HE 200x. (b) Ciliated pseudostratified squamous epithelium of oesophagus (→), HE 400x. (c) Tall, finger-like shape of mucosal projection of oesophagus, HE 40x. (d) Vascularized lamina propria of oesophagus, HE 200x.

For oesophagus, the tunica mucosa consisted of ciliated pseudostratified columnar epithelium with numerous Goblet cells especially at the first to the third segments (Figure 1(a)). The numbers of Goblet cells gradually decreased towards the posterior part (fourth and fifth segments). The cilia, which were located at the tip of the epithelium, were found abundantly at the first three segments but became lesser in number and eventually absent at the posterior part of the oesophagus (Figure 1(b)). The villi at the first part of the oesophagus were tall and broad-based with finger-like structure, which appeared star-shaped in transverse section (Figure 1(c)). The villi at the posterior segment showed gradual decrease in height and smaller base. The mean height of the mucosal projection showed no significant difference between most of the five segments of the oesophagus.

The lamina propria consisted of loose connective tissues and was highly vascularized (Figure 1(d)). However, there was no significant difference in the thicknesses of lamina propria among the segments of the oesophagus.

Tunica muscularis was arranged in two obvious layers: the inner circular muscle and the outer longitudinal muscle bundles. The thickness of both structures gradually decreased toward the posterior end of the oesophagus, obviously for the outer longitudinal muscle. Both muscular layers were significantly thicker in the first and second segments of the oesophagus than in the third to fifth segments.

### 3.2. Histological Features of the Stomach

**Figure 2:**
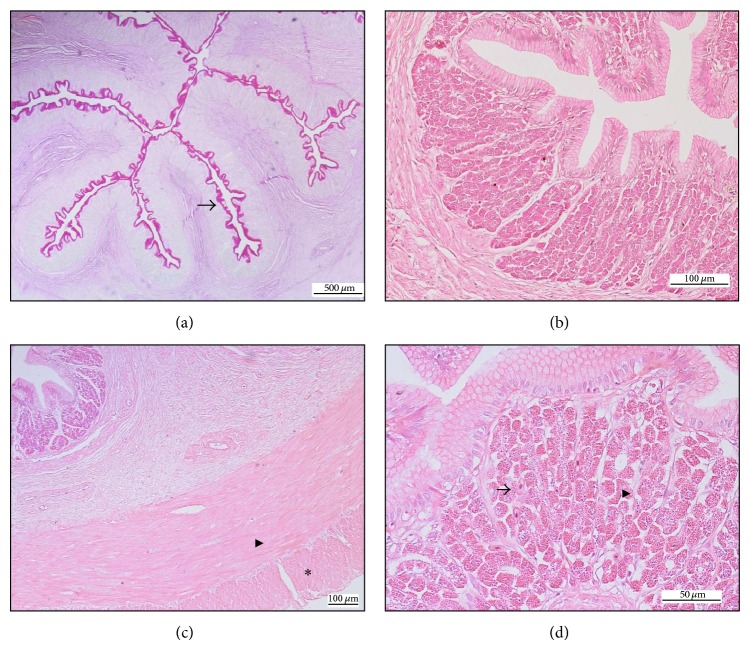
(a) Goblet cells (→) at the mucosal surface of stomach with glycogen positive stain, PAS 40x. (b) Short, sharp angle of mucosal projection of stomach, HE 200x. (c) Inner circular (▸) and outer longitudinal muscle of the stomach (∗), HE 40x. (d) Chief cells (▸) and parietal cells (→) at the gastric glands of stomach, HE 400x.

The stomach of* A. b. bicolor* consisted of the cardiac and pyloric segments. The tunica mucosa consisted of the epithelium, the basement membrane, the gastric gland, and the lamina propria. The epithelium demonstrated abrupt change from pseudostratified columnar epithelium in the oesophagus to single columnar epithelium with PAS-positive mucous cells in the stomach (Figure 2(a)). The mucosal projections were short and broad with angular edge (Figure 2(b)). However, the mean height of mucosal projection at the cardiac and pyloric stomach showed no significant difference.

The lamina propria was thick with loads of blood vessels. The cardiac segment of the stomach showed significantly thicker lamina propria than the pyloric stomach. Similarly, tunica muscularis of the stomach was composed of two layers: the inner circular muscle layer and the outer longitudinal muscle bundles (Figure 2(c)). The inner muscle at cardiac segment was significantly thicker than the pyloric segment. However, there was no significant difference in the thickness of the outer muscle layer between both segments.

Gastric glands were observed only in the stomach. The tubular-like structure was abundant and was made up of mainly chief cells with occasional parietal cells (Figure 2(d)). There was no significant difference in the thickness of gastric gland between both segments.

### 3.3. Histological Features of the Intestine

**Figure 3:**
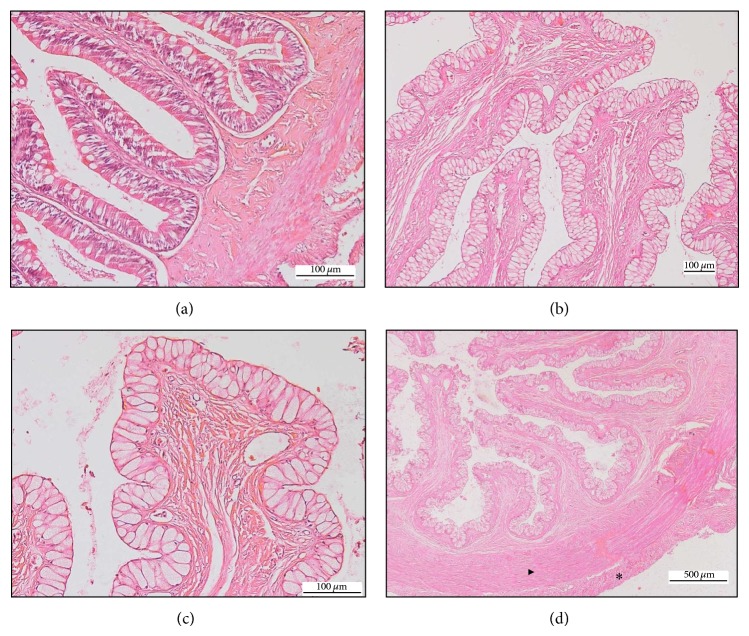
(a) The anterior part of intestine, HE 200x. (b) Goblet cells at the mucosal lining of intestine, HE 100x. (c) Vascularized villi of the intestine, HE 400x. (d) Inner circular (▸) and outer longitudinal muscle (∗) of intestine, HE 40x.

The intestine consisted of four segments, namely, the anterior, intermediate, posterior, and the rectum. The anterior intestine comprised a simple layer of columnar epithelium with prominent microvilli and scattered PAS-positive mucous cells. The intermediate and posterior intestinal mucosa showed high degree of villous folding with abundant Goblet cells (Figure 3(a)). The mucosa of the rectum was lined by a simple columnar epithelium with abundance of Goblet cells compared to the rest of the intestinal segments. There were clear histological differences that characterized the intestinal segments. The anterior segment consisted of columnar epithelium cells with brush-like border while the intermediate and posterior intestines and the rectum had no microvilli. The villous folding was thicker and the number of villi was lesser towards the end of the section, but no significant difference was noted in the height of mucosal projections among the four segments. The number of Goblet cells increased tremendously at the distal part of the tract (Figure 3(b)).

The lamina propria consisted of compact connective tissues, with numerous blood vessels especially at the tip of the villi (Figure 3(c)). The thickness of lamina propria varied without any trend throughout the intestinal tract. However, the lamina propria in the intermediate segment of the intestine was significantly thicker than in the other intestinal segments.

Tunica muscularis of the intestine included the internal circular muscle layer and the external longitudinal muscle bundles. At the last quarter of the intestinal tract, both muscles were found to be significantly thicker than the other parts of the intestine (Figure 3(d)).

## 4. Discussion

A comprehensive study of the histology of the digestive tract in* A. b. bicolor* had not been established yet although few studies were conducted on the wild and reared* A. anguilla*, but with minimal descriptions [23, 26]. Therefore, this study is crucial to understand their feeding behavior and habitat, thus providing fundamental information for further anatomical and physiological studies.

The presence of taste buds at the tip of certain mucosal projections of the anterior oesophagus of* A. anguilla* has been previously described [26, 27]. This, however, was not observed for* A. b. bicolor* in this study. Instead, this study revealed numerous cilia covering the tip of the epithelium of the oesophagus, which is believed to help in the movement of food particles and protects the oesophagus from injuries when the solid particles pass through the lumen [27]. The Goblet cells, which were abundant at anterior oesophagus, produce mucoid substance that lubricates food bolos to be easily swallowed [28]. The findings were similar to the oesophagus of* Mylio cuvieri*, which demonstrated numerous mucous cells which reacted positively to Alcian blue and PAS stains [15]. Furthermore, the oesophageal mucus is important in immunological mechanisms against bacterial infection and osmoregulatory function [29].

The height and thickness of mucosal projections and the thickness of muscularis externa of the oesophagus dramatically decreased from the anterior to the posterior part. This may be because the anterior oesophagus is the first segment to receive food bolos from the mouth and needs more contraction and movements to move the food bolos caudally during peristalsis [23]. In addition, the muscularis layer of carnivorous fish is proved to be thicker than herbivorous fish to prevent any damage or engorgement to the mucosa during swallowing preys [30].

The stomach of* A. b. bicolor* is a sac that can be stretched caudally, suggesting a feeding pattern of carnivorous fish. The organ can act as a holding area for larger bolos such as small fish that they eat. Both parts of the stomach demonstrated similar histological patterns, consisting of epithelial layer and lamina propria that contained gastric glands and muscularis externa, which is in agreement with previous study on* A. anguilla* [26]. In addition, the histological features of stomach layer of* A. b. bicolor* were also similar as observed in other carnivorous fishes, such as* Misgurnus mizolepis* [31],* Engraulis anchoita* [32], and* Dentex dentex* [33]. According to Moog and Wenger [34], cells that contain a mucoid substance, which is present at the epithelium surface, are very important for gastric protection and gastric pH control. Besides that, mucoid containing cells play an important role in complementing the digestion process [35] and was similarly observed in the stomach of* D. dentex* [33] and* Seriola dumerili* [36]. Most of the cells found in the tubular gastric glands are chief cells that secrete pepsinogen, an enzyme important for protein digestion. Few parietal cells, which secrete hydrochloric acid, can be found throughout the stomach. The stomach of herbivorous fishes,* L. niloticus* [19],* H. melanochir* [21], and* Arrhamphus sclerolepis krefftii* [37], demonstrated absence of or less hydrolysis acid, which suggested that the fishes depend on their strong pharyngeal action and teeth to rupture and grind the plant cell wall. The inner longitudinal muscle of the cardiac region was significantly thicker than pyloric region, due to its sphincter function which has some voluntary control of the food passage into the gastric sac [38]. In addition, the thick muscle fibers which arranged in two layers demonstrated the powerful trituration mechanism [19]. Nevertheless, in African butter catfish,* S. mystus*, the muscularis mucosa only found in pyloric region but absent in cardiofundic part which may be due to the primary function of the pyloric portion of stomach is mixing and pushing the food bolus distally [39]. As* A. anguilla* mainly feeds on amphipod crustaceans, insect larvae, and small fishes, this microstructure reflects the carnivorous feeding habits of the* A. b. bicolor* [40].

The intestine is a tubular organ where food from stomach passes through to start an alkaline digestion before the absorption of nutrients [41]. In fish, the length of the intestine varies and depends on the diet but is basically between 0.4 and 38 times longer than the body length. The amount of vegetal materials in diets is the main determination factor for intestinal length. Usually, herbivorous fish have longer intestine compared to carnivorous fish [16, 17, 42]. Although the intestinal segments were well differentiated histologically, no significant difference between the regions macroscopically was observed, similar to previous study on carnivorous gilthead sea bream,* Sparus aurata* [43].

The brush border epithelium of the anterior intestine of* A. b. bicolor* was found to be similar to a previous study on* A. anguilla* [23] and* Salvelinus alpinus* [44]. However, this structure was not found in other sections. According to Infante and Cahu [45], the brush border structure of marine teleost is linked to the presence of peptidase and disaccharidase enzymes important to maximize the digestion and absorption processes. Goblet cells were found throughout the intestinal tract but the number of cells increased towards the posterior intestine, a finding similar to* A. anguilla* [23]. In addition, numerous Goblet cells were found in carnivorous fishes such as* S. dumerili* [36] and* Ambassis* spp. [46]. These differences are important in the process of expulsion of feces that needs mucus substances for lubrication to ease the excretion [12]. Significantly, the herbivorous fishes, such as* L. niloticus* [22] and* L. horie* [47], demonstrated little number of Goblet cells at the posterior part of the intestine.

This study revealed that the thickness of intestinal villi gradually decreased from the anterior to the posterior section. The remnants of food particles that were not absorbed in the anterior intestine then migrate into the intermediate intestine where the absorption process continues. Since the amounts of food particles that migrate toward the intermediate and posterior intestine were lesser, the number and length of villi were significantly reduced. The result was similar to the intestine of a typical predator and a predator-facultative benthophage, which are pike,* Esox lucius*, and burbot,* Lota lota*, respectively [48]. The fishes demonstrated highest villi measurement when observed in the anterior part of the intestine, compared to the posterior part. Finally, the remaining unabsorbed food particles and wastes migrate into the rectum, waiting to be removed from the body through the anus. The villous folding and the microvilli functioned to increase the intestinal surface areas, which are important for nutrient absorption [49].

The intestinal muscularis of* A. b. bicolor* was divided into two layers: inner circular layer and outer longitudinal layer, which was in agreement with* Leporinus friderici* and* L. taeniofasciatus* [28] but was opposed to* S. dumerili* which was described to have three layers of unstriated muscle fibers [36]. It is postulated that the carnivorous fish consume various kinds of protein sources and need powerful muscle contraction at the rectal area to defecate. The propulsive contractions are caused by the muscularis externa. Because of that, the thickness of the muscle is more remarkable at the posterior part of the intestine. In contrast, the herbivorous fish which consume high fibrous contents encourage the intestinal peristalsis and less rectal muscle contraction was needed. The findings were in agreement with what has been observed in amberjack,* S. dumerili* [36], but contradicted muscularis layer of* Tilapia* spp. which demonstrated similar thickness throughout the intestine [50].

## 5. Conclusions

The present study suggested that the histological features of the gastrointestinal tract of* A. b. bicolor* were consistent with the feeding habit of a carnivorous fish. However, more studies should be carried out for deeper understanding of the digestion process and nutrient absorption of those fish.

## Figures and Tables

**Table 1:** The measurement of different features in all segments and between oesophagus, stomach, and intestine of collected *Anguilla bicolor bicolor. *

Organs	Segments	Features (*µ*m)				
		MP	LP	ICM	OLM	GG
Oesophagus	1st	1130.8 ± 72.1^abc^	113.1 ± 11.9^d^	278.1 ± 8.3^c^	229.7 ± 6.2^b^	—
	2nd	675.5 ± 40.1^d^	74.4 ± 4.9^d^	262.5 ± 12.2^c^	217.6 ± 12.9^bc^	—
	3rd	739.8 ± 25.5^cd^	123.8 ± 8.0^d^	73.7 ± 6.9^d^	128.4 ± 6.1^de^	—
	4th	795.5 ± 28.7^bcd^	71.9 ± 10.9^d^	71.6 ± 6.2^d^	87.8 ± 7.0^ef^	—
	5th	858.8 ± 59.8^bcd^	41.0 ± 2.4^d^	74.5 ± 4.5^d^	63.9 ± 3.9^f^	—
	Mean ± SEM	840.1 ± 27.9^C^	84.9 ± 5.1^B^	152.1 ± 11.8^B^	145.5 ± 8.5^B^	—

Stomach	Cardiac	1438.2 ± 134.4^a^	424.0 ± 37.9^ab^	555.5 ± 25.0^a^	151.9 ± 5.9^d^	222.8 ± 15.7^a^
	Pyloric	1146.3 ± 120.6^ab^	266.7 ± 17.3^c^	282.1 ± 8.8^c^	173.5 ± 9.6^cd^	241.5 ± 68.2^a^
	Mean ± SEM	1292.2 ± 92.8^A^	345.4 ± 25.1^A^	418.9 ± 28.5^A^	162.7 ± 5.9^B^	232.1 ± 41.9

Intestine	Anterior	1022.3 ± 122.59^bcd^	257.9 ± 21.4^c^	235.9 ± 4.2^c^	120.1 ± 8.6^de^	—
	Intermediate	1160.3 ± 92.8^ab^	480.9 ± 32.8^a^	237.9 ± 8.2^c^	117.9 ± 8.2^def^	—
	Posterior	964.0 ± 112.0^bcd^	302.9 ± 22.7^c^	287.8 ± 10.6^c^	212.9 ± 13.9^bc^	—
	Rectum	895.8 ± 68.9^bcd^	333.5 ± 18.9^bc^	379.9 ± 23.4^b^	469.7 ± 29.7^a^	—
	Mean ± SEM	1010.6 ± 50.9^B^	343.8 ± 16.2^A^	285.4 ± 10.1^B^	230.2 ± 20.5^A^	—

MP: height of mucosal projection; LP: thickness of lamina propria; ICM: thickness of inner circular muscle; OLM: thickness of outer longitudinal muscle; and GG: thickness of gastric gland. Data are presented in mean ± (standard error of mean) SEM from 28 collected samples. For comparison, features in different organs and segments, the same superscript in uppercase letter within columns and lowercase letter within rows, respectively, are not significantly different.
